# Gastrointestinal bleeding in hepatocellular carcinoma under systemic therapy: Real-world incidence and clinical predictors

**DOI:** 10.1016/j.clinsp.2026.101155

**Published:** 2026-09-17

**Authors:** Leonardo G Da Fonseca, Lucas Takeshi Ikeoka, Eduardo Kim Sampaio, Thamires Haick Martins da Silveira, Victor Junji Yamamoto, Marina Acevedo Zarzar de Melo, Paulo Marcelo Hoff, Jorge Sabbaga

**Affiliations:** Department of Oncology, Instituto do Câncer do Estado de São Paulo (ICESP), Faculdade de Medicina da Universidade de São Paulo (FMUSP), São Paulo, SP, Brazil

**Keywords:** Hepatocellular carcinoma, Gastrointestinal bleeding, Portal hypertension, Cirrhosis, Risk factors

## Abstract

**Objective:**

Gastrointestinal (GI) bleeding is a major complication in patients with advanced Hepatocellular Carcinoma (HCC), particularly among those with underlying portal hypertension. Although systemic therapy has improved survival outcomes, real-world data regarding the incidence, predictors, and clinical impact of GI bleeding remain limited. This study aimed to evaluate the incidence, risk factors, and prognostic implications of GI bleeding in patients with advanced HCC receiving sorafenib.

**Methods:**

This retrospective cohort study included 439 patients with advanced HCC between 2009 and 2025. Time-to-event analyses were performed from treatment initiation. The cumulative incidence of GI bleeding and bleeding-related mortality was estimated using competing-risk methods. Incidence rates were calculated per 100 Person-Years (PY), and adjusted incidence rate ratios were estimated using multivariable Poisson regression. Predictors of GI bleeding were further evaluated using multivariable logistic regression.

**Results:**

Baseline endoscopy was performed in 56% of patients, of whom 58% had evidence of portal hypertension. The incidence rate of GI bleeding was 5.6 per 100 PY (95% Confidence Interval [95% CI 4.6–6.8]), with cumulative incidence rates of 10.57% (95% CI 7.85–14.16) at 6-months and 23.06% (95% CI 18.60–28.38) at 12-months. Esophagogastric varices, splenomegaly, vascular invasion, and prior GI bleeding were independently associated with increased bleeding risk. Based on these predictors, a simple additive risk score ranging from 0 to 4 was developed and demonstrated progressive increases in predicted bleeding probability across risk categories, supporting its potential utility for clinical risk stratification. GI bleeding was associated with poor outcomes, with a median survival time of 11-days after the event and a bleeding-related mortality rate of 75.5%.

**Conclusion:**

GI bleeding is a frequent and highly lethal complication in patients with advanced HCC. These findings highlight the importance of risk assessment, endoscopic evaluation, and preventive strategies in this population.

## Highlights

• GI bleeding occurred in 24.1% of HCC patients during systemic therapy.
• GI bleeding reached 23.1% cumulative incidence at 12-months.
• Varices, splenomegaly and vascular invasion predicted bleeding risk.
• A simple 4-point score stratified GI bleeding risk during treatment.
• GI bleeding was associated with 75.5% bleeding-related mortality.

## Introduction

Hepatocellular Carcinoma (HCC) is a leading cause of cancer-related death, largely due to frequent coexistence with chronic liver disease and the high proportion of patients diagnosed at the advanced stages^1^ Systemic therapy has become essential in the management of advanced HCC, with multiple first-line options now available, including immune checkpoint inhibitor combinations, such as atezolizumab plus bevacizumab, durvalumab plus tremelimumab and nivolumab plus ipilimumab, as well as multikinase inhibitors, such as sorafenib and lenvatinib^2^, ^3^, ^4^, ^5^ Therapy selection is guided not only by efficacy but also by safety, particularly in patients with underlying cirrhosis, who have an increased risk of treatment-related complications.

Cirrhosis is frequently accompanied by portal hypertension, which can predispose patients to clinically significant complications, including decompensation and gastrointestinal (GI) bleeding from esophageal or gastric varices^1^ The risk of variceal bleeding represents a notable concern in the management of HCC, as it may limit treatment tolerability and negatively impact survival. Furthermore, multikinase inhibitors (for example, sorafenib) and bevacizumab may increase the risk of severe bleeding events^6^ While controlled clinical trials have reported low rates of high-grade upper GI bleeding in HCC patients receiving sorafenib (2%) or atezolizumab plus bevacizumab (1.8%‒2.4%), real-world data suggest a higher incidence, with bleeding reported in 8%‒12% of patients, mainly in those with high-risk features such as portal vein tumor invasion.^7^, ^8^, ^9^

The unprecedented advances in HCC management have provided long-term tumor control in a proportion of patients. However, cirrhosis complications still pose a competing risk of mortality^1^ Therefore, reports on the incidence and predictors of cirrhosis-related complications, such as upper GI bleeding, are becoming more critical in HCC management. Understanding this context can assist with baseline risk assessment, prophylactic strategies and treatment decisions. To explore this, the present retrospective cohort study was conducted to evaluate the real-world clinical practice, incidence, predictors and clinical outcomes of upper GI bleeding in patients with advanced HCC undergoing systemic therapy.

## Material and methods

### Study design and patient population

A database of patients with HCC treated consecutively at the Instituto do Cancer do Estado de São Paulo (São Paulo, Brazil) from July 2009 to June 2025 was retrospectively reviewed. Patients were included in the present study if they had a diagnosis of HCC based on radiological or histological criteria and had started first-line systemic therapy according to local policy. Only patients with intermediate (Barcelona Clinic Liver Cancer stage B [BCLC-B]) or advanced (BCLC—C) HCC not suitable for locoregional therapies were considered. The study protocol complied with the STROBE guidelines and adhered to international ethical standards and Good Clinical Practice.^10^ The local Ethics Committee (Ethical Commission, Hospital das Clinicas ‒ University of Sao Paulo School of Medicine) approved the study and the requirement for informed consent was waived due to the retrospective design (approval report 8.274.176). Data were collected exclusively from routinely generated medical records after clinical care had already occurred, without protocol-driven interventions.

### Data collection

Data were collected from medical records, including age, sex, Eastern Cooperative Oncology Group (ECOG) performance status, etiology of liver disease, BCLC stage, serum laboratory parameters and radiological findings. Vascular invasion was defined on baseline images as a filling defect that occluded the vessel in the portal venous phase, with clear arterial-phase enhancement. Splenomegaly was defined as a splenic craniocaudal length measurement exceeding 12 cm based on radiological assessment. Esophageal varices were classified according to the North Italian Endoscopic Club criteria: Size and location (*F*, Graded 0‒3) and red wale marks^11^ Endoscopic examinations performed within 12-months before treatment initiation were accepted as baseline evaluations because this reflected routine clinical practice during the study period. Data were last updated in January 2026.

### Treatment and follow-up

Throughout the study period (2009–2025), sorafenib remained the only routinely available first-line systemic therapy for advanced HCC in the Brazilian public health system at our institution^12^ Therefore, all patients included in this cohort received first-line sorafenib. Sorafenib was administered orally at an initial dose of 400 mg twice daily, with dose adjustments based on the occurrence of adverse events. Treatment continued until symptomatic or radiological progression, intolerance or death. Clinical and laboratory assessments were performed at baseline and monthly. Radiological evaluations were performed every 2‒3 months.

### Bleeding events and follow-up definitions

GI bleeding was defined as any episode of upper GI hemorrhage originating from the esophagus, stomach or duodenum. Bleeding events were classified as upper GI bleeding when one of the following criteria was met: i) Endoscopically confirmed bleeding from the upper GI tract, including variceal or non-variceal sources (including hypertensive gastropathy or peptic ulcer disease); ii) The presence of large esophagogastric varices with blood in the stomach, in the absence of an alternative identifiable bleeding source; or iii) A clinically documented episode of hematemesis and/or melena in a patient with established portal hypertension, even when endoscopy was unavailable or inconclusive. Bleeding was managed according to the Baveno International Consensus Criteria.^13^

Bleeding events during treatment were analyzed using time-to-event methods. The time at risk was defined as the period from treatment initiation to the first bleeding event or the date of the last follow-up. Patients without bleeding events were censored at their last clinical assessment. Because death occurring before an upper GI bleeding event precludes the subsequent observation of bleeding during follow-up, death was treated as a competing event in the cumulative incidence analyses. Cumulative incidence was calculated for the overall cohort and for clinically relevant subgroups, including those according to Child-Pugh class, Albumin-Bilirubin (ALBI) score and BCLC stage. Bleeding incidence rates were calculated using person-time methods and are expressed as events per 100 Person-Years (PY).

Cumulative mortality due to bleeding was estimated from the start of systemic therapy using competing-risk methods, treating non-bleeding-related deaths as competing events. Adjusted Incidence Rate Ratios (IRRs) were calculated using multivariable Poisson regression models with PY as the offset, including clinically relevant covariates.^14^

A bleeding risk score was derived from the final multivariable logistic regression model. Independent predictors (esophagogastric varices, splenomegaly, vascular invasion, and prior GI bleeding) were assigned equal weights (1-point each), resulting in a total score ranging from 0 to 4. Individual predicted probabilities of upper GI bleeding were estimated using the fitted logistic model, and the mean predicted probability for each score category was calculated and reported with the corresponding standard deviation (SD). To facilitate clinical interpretation, scores were further grouped into low (0–1), intermediate (2), and high (≥ 3) risk categories.

### Statistical analysis

Quantitative variables are reported as medians and interquartile ranges (IQRs), while categorical variables are reported as counts and percentages. Categorical variables were compared using Fisher’s exact test. Factors associated with bleeding during treatment were evaluated using multivariable logistic regression. Candidate predictors included Child-Pugh class, ALBI score, BCLC stage, presence of vascular invasion, previous GI bleeding events, prophylaxis and baseline esophagogastric varices. A backward stepwise selection procedure was applied, removing variables with p > 0.05, to derive the final model of independent predictors. Model calibration was assessed using the Hosmer-Lemeshow goodness-of-fit test (χ^2^ statistic), with p > 0.05 indicating adequate agreement between observed and predicted event rates^15^ The discriminatory performance of the proposed bleeding risk score was assessed by calculating the Area Under the receiver operating Characteristic Curve (AUC). Internal validation was performed using bootstrap resampling with 1000 iterations to estimate the optimism-corrected discriminatory performance of the model. All statistical analyses were performed using Stata version 15.1 (StataCorp LP). Two-sided p < 0.05 was considered to indicate a statistically significant difference.

## Results

### Baseline characteristics

All 439 patients included in the present study received first-line sorafenib according to institutional and Brazilian public health system protocols during the study period. Most patients were male (73.3%; n = 322), and hepatitis C was the most common background liver disease (50.3%; n = 221). At baseline, 82.7% of patients were classified as Child-Pugh A and 73.6% were in the BCLC—C stage. The median age was 63.1-years (range: 31.5‒83.4). Macrovascular invasion was observed in 41.7% of patients and extrahepatic metastasis in 40.5%. Previous local or regional treatments, mainly transarterial chemoembolization, were received by 43.3% of patients (Table 1).

**Table 1:** Baseline clinical and laboratory features.

Baseline characteristics	n = 439
Median age (IQR)	63.1 (55.5‒69.5)
Sex	
Male, n (%)	322 (73.3%)
Female, n (%)	117 (26.6%)
Chronic liver disease etiology^a^	
No hepatopathy, n (%)	11 (2.5%)
Hepatitis C, n (%)	221 (50.3%)
Hepatitis B, n (%)	61 (13.9%)
Alcohol, n (%)	115 (26.1%)
MASLD, n (%)	45 (10.3%)
Previous treatment^b^	
Transplant, n (%)	28 (6.4%)
Resection, n (%)	53 (12.1%)
Ablation, n (%)	44 (10%)
TACE, n (%)	190 (43.3%)
No previous treatment, n (%)	207 (47.2%)
Child-Pugh	
A	363 (82.7%)
B	76 (17.3%)
ALBI score	
1, n (%)	175 (39.8%)
2, n (%)	249 (56.7%)
3, n (%)	15 (3.4%)
BCLC stage	
B, n (%)	116 (26.4%)
C, n (%)	323 (73.6%)
Macrovascular invasion, n (%)	183 (41.7%)
Extrahepatic spread, n (%)	178 (40.5%)
ECOG Performance status, n (%)	
0‒1, n (%)	391 (89.1%)
2, n (%)	48 (10.9%)
Alpha-fetoprotein, median (IQR)	211.7 (17‒3607)
Platelets × 10^3^, median (IQR)	142 (85‒210)

IQR, Interquartile Range; MASLD, Metabolic-Associated Steatotic Liver Disease; TACE, Transarterial Chemoembolization; BCLC, Barcelona Clinic Liver Cancer; ECOG, Eastern Cooperative Oncology Group.

a n = 4 patients had Hepatitis C plus Alcohol.

b 83 patients received more than 1 previous treatment.

### Bleeding risk assessment

Baseline endoscopy was performed in 245 (55.8%) patients, with a median time of 5.6-months (IQR 1.3‒12.5) before the initiation of systemic treatment. No significant differences were observed between patients who underwent endoscopy and those who did not with respect to Child-Pugh score (p = 0.548), splenomegaly (p = 0.104), BCLC stage (p = 0.08) or vascular invasion (p = 0.563). To analyze changes in practice over time, we compared the endoscopy referral rate across two periods. From 2009‒2015, 46.1% (90/195) of patients underwent baseline endoscopy, whereas 63.5% (155/244) underwent endoscopy from 2016‒2025 (p < 0.001). No significant endoscopic findings were reported in 103 (42.0%) patients, while 74 (30.2%) had small esophageal varices, 40 (16.3%) had medium-sized esophageal varices, 10 (4.1%) had large esophageal varices, 12 (4.9%) showed red spots, 89 (36.3%) had hypertensive gastropathy and 21 (8.6%) had gastric varices. Previous GI bleeding events were reported in 68 (15.5%) patients. Primary or secondary prophylaxis was administered to 170 (38.7%) patients, mainly beta-blockers (n = 110; 64.7%) or banding (n = 52; 30.6%), while 8 (4.7%) patients received a combination of banding and beta-blockers. No temporal changes were observed between the use of prophylaxis across the period between 2009‒2015 and 2016‒2025 (p = 0.279). Regarding other signs of portal hypertension, splenomegaly was reported for 231 (52.6%) patients. The median platelet count was 142,000/mm^3^ (IQR 85,000‒210,000), and 80 (18.2%) patients had a platelet count < 75,000/mm^3^.

### Bleeding events

**Fig. 1:**
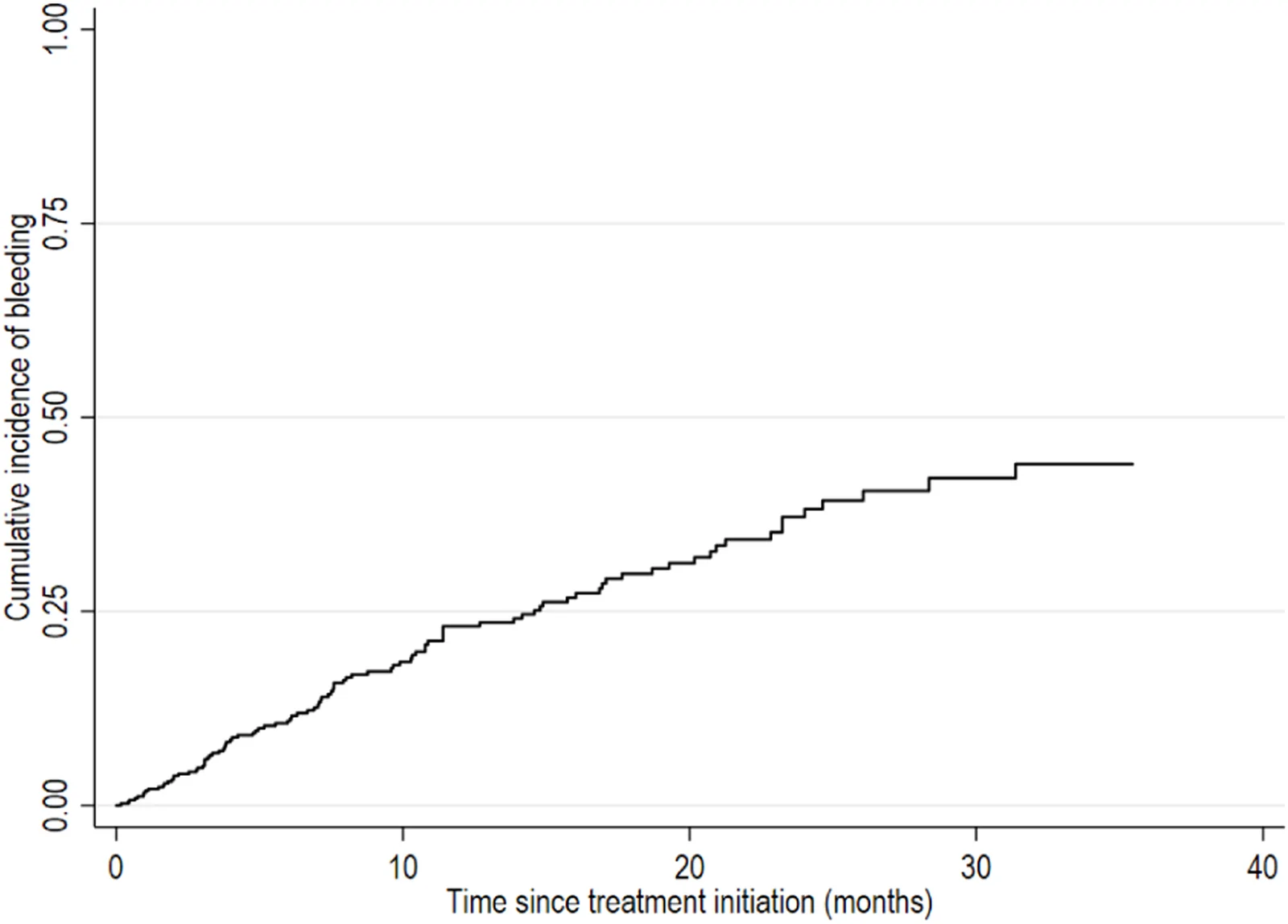
Cumulative incidence of bleeding during sorafenib treatment, accounting for death as a competing event.

During a median follow-up time of 9.1-months (IQR 3.9‒18.8) after starting systemic treatment, 106 (24.1%) patients experienced upper GI bleeding events. The median follow-up for patients who had an upper GI bleeding event was 8.3-months (IQR 4.1‒14.7), while the median follow-up for patients who had not presented with an upper GI bleeding event was 9.2-months (IQR 3.9‒19.7). Among these 106 patients, bleeding source confirmation by endoscopy was available for 93 (87.7%), while 13 (12.3%) were diagnosed based on clinical criteria when endoscopy was unavailable or inconclusive. Of the 93 patients with confirmatory endoscopy, the source of bleeding was variceal in 78, while 15 were described as having bleeding due to hypertensive gastropathy. Among the patients who presented with upper GI bleeding, 69 (65.1%) had undergone a prior endoscopy, whereas 37 (34.9%) had not. The incidence rate of GI bleeding was 5.6 per 100 PY (95% CI 4.6‒6.8), after a median follow-up time of 489.1 PY. The cumulative incidence of upper GI bleeding was 4.8% (95% CI 3.1‒7.4%) at 3-months, 10.6% (95% CI 7.9‒14.2%) at 6-months and 23.1% (95% CI 18.6‒28.4%) at 12-months (Table 2 and Fig. 1).

**Table 2:** Cumulative incidence of bleeding during treatment at 1-, 3-, 6-, 9-, 12-, 18-, and 24-months, with corresponding 95% Confidence Intervals (95% CI), estimated using competing-risk methods.

Time	Cumulative Incidence function	95%CI
1-month	0.0163	0.0078‒0.0339
3-months	0.0483	0.0314‒0.0739
6-months	0.1057	0.0785‒0.1416
9-months	0.1724	0.1356‒0.2178
12-months	0.2306	0.1860‒0.2838
18-months	0.2985	0.2460‒0.3606
24-months	0.3715	0.3069‒0.4448

Subgroup analyses showed differences in upper GI bleeding risk based on baseline liver function, tumor stage and portal hypertension findings. Patients with impaired liver function had higher bleeding rates. Specifically, patients with Child-Pugh B had upper GI bleeding at a rate of 73.1 per 100 PY, compared with 17.8 per 100 PY in patients with Child-Pugh A. Similarly, patients with an ALBI score of 1 had a bleeding incidence of 13.4 per 100 PY, versus 28.4 per 100 PY in those with an ALBI score of 2 (Table 3).

**Table 3:** Incidence of bleeding during treatment by clinically relevant subgroups. The table reports the number of bleeding events, total follow-up time expressed as Person-Years (PY), and incidence rates per 100 PY with corresponding 95% Confidence Intervals (95% CI). Incidence Rate Ratios (IRR) with 95% CI and p-values were estimated using Poisson regression, with the reference category defined within each subgroup.

Subgroup	Events	Person-years	Incidence per 100PY (95% CI)	IRR (95% CI); p
Child-Pugh A	81	454.6	17.8 (14.1‒22.1)	Reference
Child-Pugh B	25	34.2	73.1 (47.3‒107.9)	4.10 (2.6‒6.4); p < 0.001
ALBI 1	31	232.2	13.4 (9.1‒19)	Reference
ALBI 2	67	236.2	28.4 (22.0‒36.0)	2.1 (0.9‒3.2); p = 0.09
BCLC B	24	157.1	15.3 (9.8‒22.7)	Reference
BCLC C	82	322.0	25.5 (20.3‒31.6)	1.8 (1.1‒2.7); p = 0.03
Vascular invasion	56	295.8	18.9 (14.3‒24.6)	Reference
No vascular invasion	52	339.2	15.3 (11.4‒20.1)	0.8 (0.7‒1.8); p = 0.306
No Gastroesophageal varices	10	83.3	12.0 (5.8‒22.1)	Reference
Gastroesophageal varices	40	110.2	36.3 (25.9‒49.4)	3.0 (1.5‒6.4); p = 0.02
No previous bleeding	72	427.8	16.8 (13.2‒21.2)	Reference
Previous bleeding	34	56.7	60 (41.5‒83.8)	3.6 (2.3‒5.5); p < 0.001
No splenomegaly	25	235.1	10.6 (6.9‒15.7)	Reference
Splenomegaly	78	237.1	32.9 (26.0‒41.1)	3.1 (0.9‒4.9); p = 0.09

Reference category for each subgroup is the first level listed (Child-Pugh A, ALBI 1, BCLC B, no varices, no prior bleeding, no splenomegaly).

**Fig. 2:**
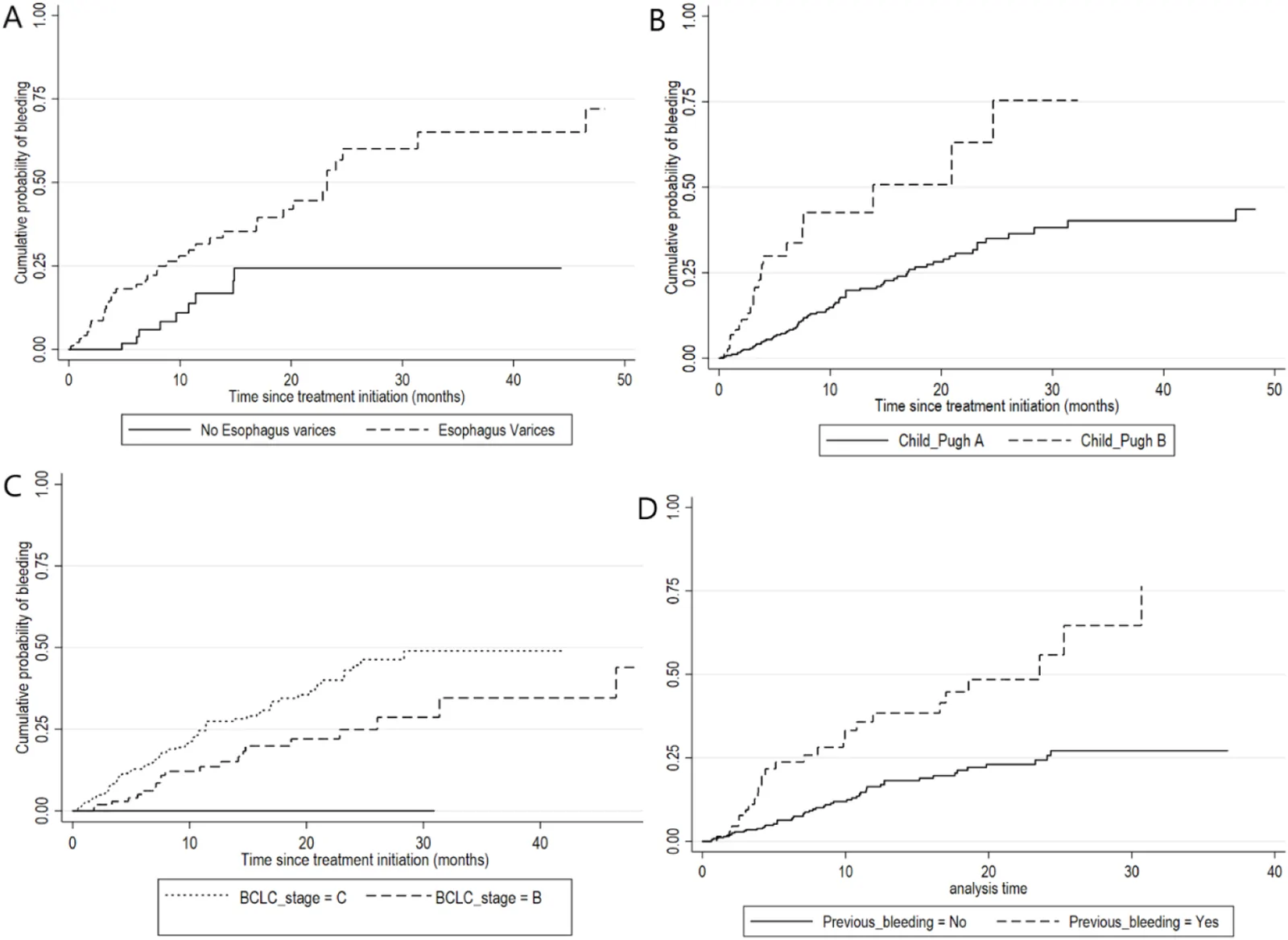
Cumulative incidence of GI bleeding, accounting for death as a competing event. Curves are shown according to clinically relevant subgroups: (A) Esophagogastric varices (yes vs. no, upper left panel), (B) Child–Pugh class (A vs. B, upper right panel), (C) BCLC stage (B vs. C, bottom left panel), and (D) Previous history of bleeding (yes vs. no, bottom right panel). The y-axis represents the cumulative incidence of bleeding, and the x-axis represents months since treatment initiation.

Tumor stage was also associated with upper GI bleeding frequency. Patients with BCLC—C had an incidence rate of 25.5 per 100 PY, compared with 15.5 per 100 PY for BCLC-B. The presence of vascular invasion increased the incidence of upper GI bleeding to 18.9 per 100 PY, whereas patients without vascular invasion had an incidence of 15.3 per 100 PY. Finally, patients with baseline esophageal or gastric varices had a higher upper GI bleeding incidence (36.3 per 100 PY) than those without varices (12.0 per 100 PY) (Fig. 2).

Adjusted IRR confirmed that impaired hepatic function (as defined by the Child-Pugh score), advanced tumor stage (according to the BCLC staging system), baseline varices and prior bleeding history before systemic treatment were associated with a higher incidence of upper GI bleeding during treatment (Table 3).

To evaluate the association between prophylaxis and the occurrence of upper GI bleeding, we performed a logistic regression adjusted for high-risk endoscopic findings (large varices or red wale signs). This adjustment was intended to mitigate confounding, as patients with high-risk features are more likely to develop bleeding. Among patients with high-risk endoscopic findings, primary prophylaxis was associated with reduced odds of bleeding (OR = 0.65; 95% CI 0.52–0.80; p = 0.019), as was secondary prophylaxis (OR = 0.79; 95% CI 0.66–0.90; p = 0.015). Due to the limited granularity of endoscopic reports in the present retrospective cohort and the small size of each subgroup, stratification by variceal size or red wale signs could only be descriptive. The 6-month cumulative risk of upper GI bleeding was 37.5% (95% CI 13.9‒77.1%) for patients with large esophageal varices and 60.0% (95% CI 24.7‒94.8%) for those with red spots. For patients with small-sized varices, the 6-month risk of upper GI bleeding was 28.1% (95% CI 21.2‒61.9%).

### Bleeding-related deaths

**Fig. 3:**
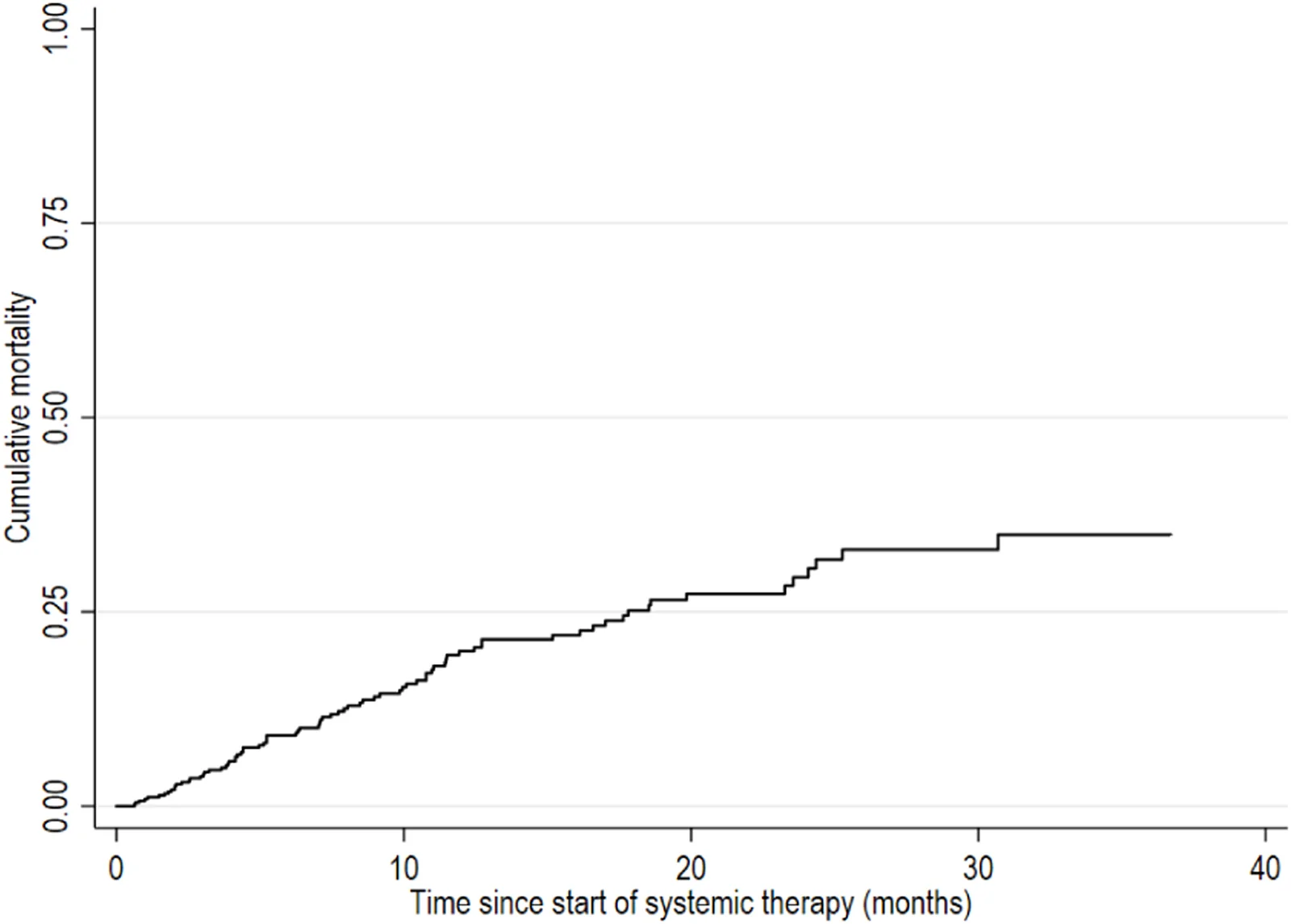
Cumulative mortality from the start of systemic therapy. The Y-axis represents cumulative mortality; the X-axis represents months since treatment initiation.

At the last follow-up, 381 patients had died. Bleeding-related deaths were reported in 80 patients, accounting for 75.5% of those who experienced an upper GI bleeding event. The median overall survival (OS) time for patients who presented with upper GI bleeding events was 8.5months (95% CI 6.8‒10.9), which was significantly lower than that for patients who did not experience bleeding, with a median OS time of 9.8months (95% CI 8.7‒11.3) and an HR of 1.3 (95% CI 1.11‒1.6; log-rank, p = 0.02). The median OS time after a bleeding event was 11-days (95% CI 6‒16 days). The proportion of patients with an upper GI bleeding event and death related to bleeding was significantly higher in those with BCLC—C compared with BCLC-B (Fisher’s exact test, p = 0.03), while no significant differences were observed when analyzing other variables such as vascular invasion (p = 0.071), splenomegaly (p = 0.484), Child-Pugh class (p = 0.097) and previous use of prophylaxis (p = 0.672). The cumulative mortality rate caused by upper GI bleeding from the start of systemic therapy was 0.9% (95% CI 0.0‒2.4%) at 1-month, 3.8% (95% CI 2.3‒6.2%) at 3-months, 9.1% (95% CI 6.5‒12.5) at 6-months and 19.9% (95% CI 15.7‒25.1%) at 12-months (Fig. 3).

### GI bleeding prediction

In the multivariable logistic regression model using backward stepwise selection, esophagogastric varices (Odds Ratio [OR = 1.3]; 95% CI 1.1‒1.7; p = 0.024), the presence of splenomegaly (OR = 2.4; 95% CI 1.2‒4.8; p = 0.011), vascular invasion (OR = 2.1; 95% CI 1.1‒3.9; p = 0.019) and past bleeding events before systemic treatment (OR = 2.7; 95% CI 1.3‒5.5; p = 0.005) were independently associated with upper GI bleeding after systemic treatment initiation. Other candidate variables, including ALBI score, BCLC stage, and platelet count < 100,000 were excluded from the selection process as their P-values were > 0.05. The Hosmer-Lemeshow test indicated a good model fit (χ^2^ = 4.18, p = 0.840), suggesting that the predicted probabilities were consistent with the observed bleeding events.

### Bleeding risk stratification

To further explore the clinical applicability of the multivariable model, a simple additive risk score was constructed based on the four independently associated predictors (esophagogastric varices, splenomegaly, vascular invasion, and prior GI bleeding). Each variable contributed 1-point, resulting in a total score ranging from 0 to 4.

**Fig. 4:**
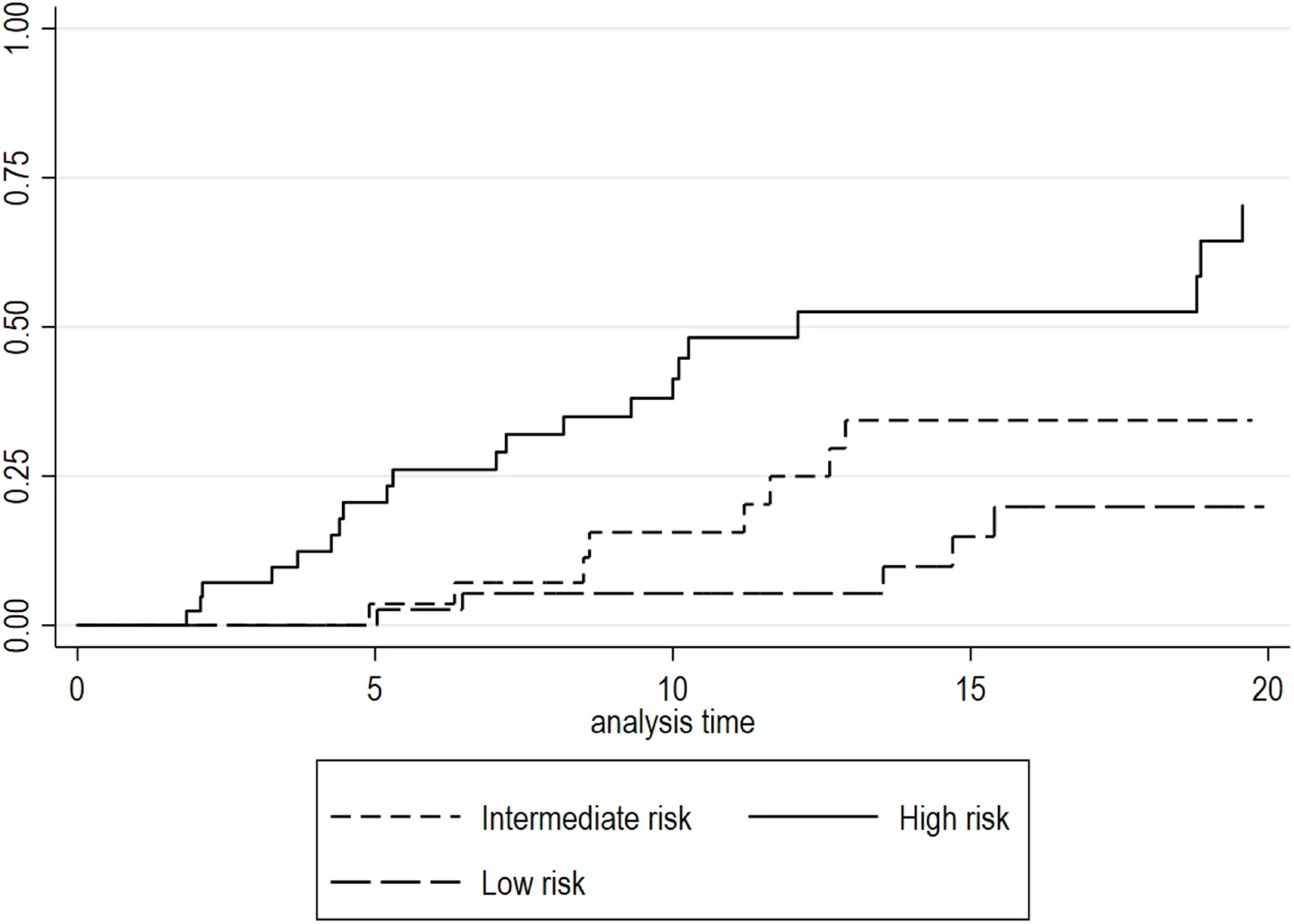
Cumulative incidence of bleeding scored according to esophagogastric varices, splenomegaly, vascular invasion, and prior GI bleeding. Each variable contributed 1-point: low risk (0–1 points), intermediate risk (2-points), and high risk (≥3-points).

The predicted probability of upper GI bleeding increased progressively with higher risk scores, as estimated from the logistic regression model. Patients with a score of 0 had a mean predicted probability of 22.4% (SD = 5.0%), which increased to 24.0% (SD = 6.2%) with a score of 1, 28.1% (SD = 7.2%) with a score of 2, 29.5% (SD = 7.4%) with a score of 3, and 33.6% (SD = 4.5%) among those with all four risk factors. The cumulative incidence of upper GI bleeding across scores is shown in Fig. 4.

Although the incremental increase in risk was modest between adjacent categories, the overall trend supports a gradual and clinically meaningful increase in bleeding probability with greater accumulation of risk factors. These findings indicate that the proposed score has the ability to stratify patients according to bleeding risk, with those in higher score categories exhibiting a substantially increased probability of events during follow-up.

**Fig. 5:**
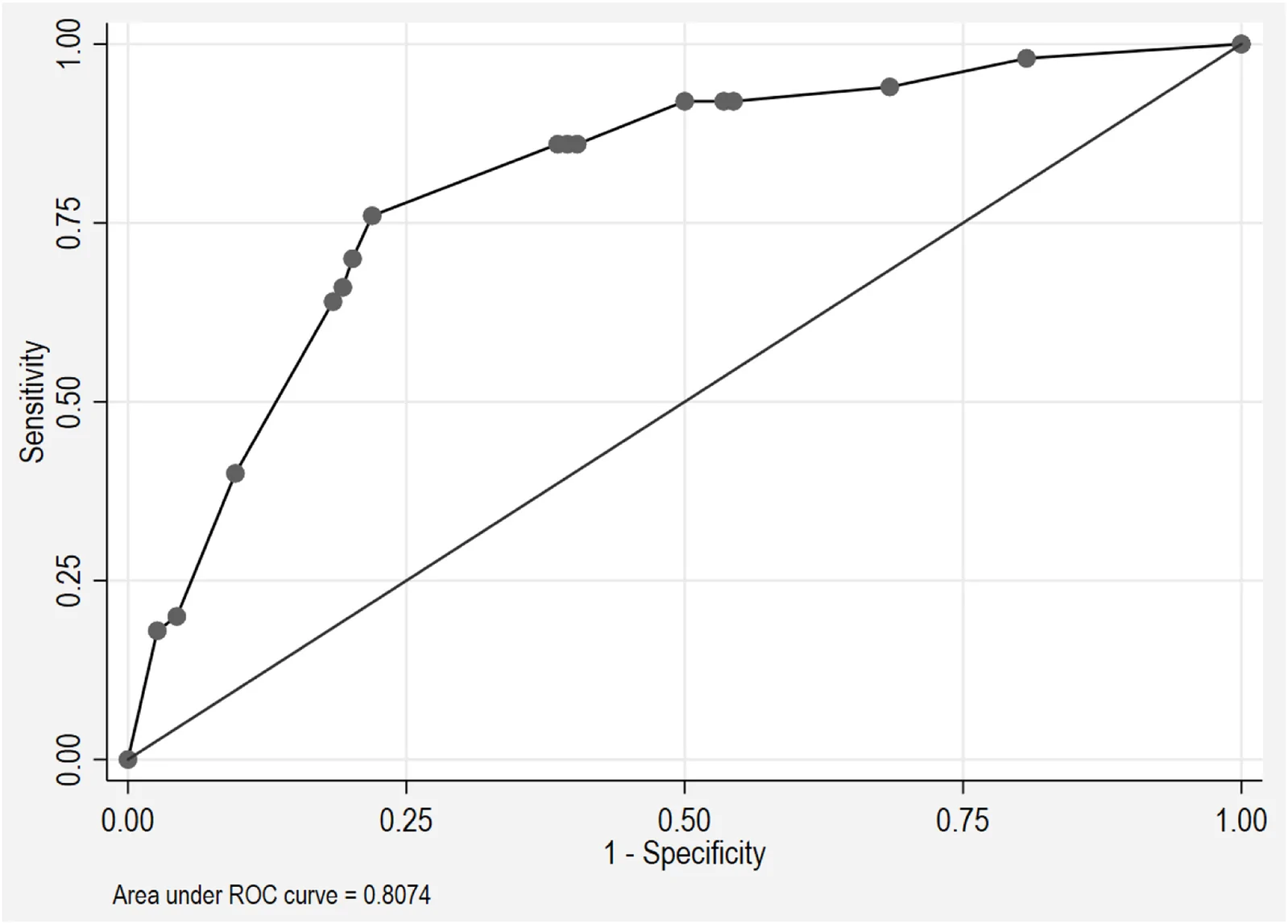
Receiver Operating Characteristic (ROC) curve for the proposed four-point bleeding risk score. The ROC curve demonstrates the discriminatory performance of the simplified bleeding risk score for predicting upper gastrointestinal bleeding.

To facilitate clinical interpretation, patients were grouped into three categories: low risk (0–1 points), intermediate risk (2-points), and high risk (≥ 3-points). The corresponding mean predicted probabilities were approximately 23.6%, 28.1%, and 31.0%, respectively, supporting the use of this classification for risk-adapted management strategies. The model showed acceptable discrimination (AUC = 0.81 [95% CI 0.74‒0.88]) (Fig. 5). Internal validation using 1000 bootstrap resamples demonstrated similar discrimination (bootstrap-corrected AUC = 0.81), suggesting robustness of the model.

## Discussion

The present study assessed the incidence, risk factors and outcomes of upper GI bleeding in a large group of patients with advanced HCC receiving systemic therapy. The results showed that upper GI bleeding was a common and serious complication, with the mortality rate reaching 75.5%. Notably, cumulative incidence analyses accounting for competing risks revealed a steady increase over time, reaching 23.1% at 12-months, underscoring the need for ongoing monitoring during treatment. The present real-world study reported that ∼44% of patients who initiated systemic treatment had no prior risk stratification based on endoscopy, and there was no difference in liver function and vascular invasion among patients who did not undergo endoscopy. Because of the retrospective nature of the study, the specific reasons for omission of baseline endoscopy could not be reliably determined from medical records. Potential explanations include differences in referral patterns, access to endoscopy, clinical urgency, and historical practice changes during the study period. Among those with baseline endoscopy data, 58% showed findings indicative of portal hypertension. Additionally, 34% of the patients who presented with upper GI bleeding events had not undergone prior endoscopy.

Over the past years, particularly following the approval of atezolizumab-bevacizumab combination therapy, data have highlighted safety concerns of this regimen, underscoring the need to address bleeding risk in patients with HCC. This likely reflects the higher rate of endoscopy referrals observed in our cohort in recent years. The IMbrave150 trial required that all patients undergo an endoscopy within 6-months before treatment and that prophylaxis be administered for GI varices^3^ However, prior phase III studies performed before 2018, such as the SHARP^16^ and REFLECT^2^ trials, were less stringent in their inclusion criteria. In a real-world study of patients with HCC treated with lenvatinib between 2014 and 2022, 34.4% of patients had not undergone prior endoscopy within the past 6-months^17^ In the observational AB-real study, which included patients treated with atezolizumab-bevacizumab, only 53% had undergone prior endoscopies^7^ Differences between trial-based safety estimates and routine clinical practice likely reflect stricter eligibility criteria, mandatory baseline screening/prophylaxis strategies in some trials and the exclusion of patients with recent bleeding or more advanced liver dysfunction, whereas real-world cohorts include broader clinical phenotypes and less standardized risk stratification. The role of endoscopy, particularly in high-risk patients, was also highlighted by Allaire et al^18^ who showed that non-invasive parameters such as liver stiffness and platelet count may not reliably exclude large esophageal varices in a study of 185 patients with HCC. In this study, even among patients with preserved liver function and across different BCLC stages, a substantial proportion of patients still had high-risk varices despite non-invasive assessment. Altogether, the results of the present study aligned with these findings, underscoring the need for careful, individualized risk assessment and, in most cases, endoscopic evaluation before initiating systemic therapy.

The present cohort included subgroups of patients that have not previously been included in clinical trials, such as those with Child-Pugh B, ECOG performance status 2 and thrombocytopenia, which may explain the higher bleeding incidence observed compared with clinical trials. Other real-world studies with a diverse HCC population also showed a high incidence of bleeding. For instance, Parikh et al^19^ analyzed 904 patients with HCC under systemic treatment (mostly sorafenib) and observed variceal bleedings among 15.3% of the 175 patients with known esophagogastric varices and bleeding-related emergency room visits or hospitalizations due to bleeding events in 15.6% of all patients.

Consistent with prior reports, we observed that impaired hepatic function and markers of portal hypertension have emerged as strong predictors of GI bleeding after systemic treatment. Studies have shown that patients with Child-Pugh B cirrhosis (vs. Child-Pugh A) or baseline esophagogastric varices experience significantly higher cumulative incidence rates^8^^,^^9^^,^^17^^,^^19^, ^20^, ^21^ Similarly, BCLC—C (vs. BCLC-B) also experience higher incidence rates, highlighting that both tumor-related and portal hypertension-related factors increase the risk of upper GI bleeding. Because most confirmed bleeding events were variceal or portal hypertension-related, risk factors were interpreted primarily in the context of portal hypertension severity. These findings align with the known pathophysiology of the combination of portal hypertension and tumor progression.

In the present study, patients who experienced an upper GI bleeding event had a significantly reduced OS time, with a median OS time of only 11-days after the event. This extremely poor post-bleeding survival time underscores that GI bleeding in this setting often represents a terminal event. In the IMbrave150 trial, for example, the median OS following a first any-grade GI bleeding event was 3.3-months (IQR 0.4–6.7)^3^ Potential contributors include advanced tumor burden, because BCLC—C patients were associated with a higher risk of bleeding-related deaths and were the predominant stage included in the present study.

The multivariable logistic regression model identified key predictors of upper GI bleeding that could inform clinical decision-making and preventive measures. The presence of esophagogastric varices, splenomegaly, vascular invasion and prior bleeding events was independently associated with an increased risk of upper GI bleeding. These findings were consistent with other observational studies. For instance, Larrey et al^22^ found that a history of acute variceal bleeding increased the risk of bleeding during systemic treatment for HCC (HR = 10.58; p = 0.03) in a cohort of 43 patients. Zhang et al^23^ developed a risk model to predict variceal bleeding using variables significantly associated with bleeding events, including variceal bleeding, tumor size, macrovascular invasion, FIB-4 index and prior bleeding. Lim et al. ^9^ also highlighted a higher risk of bleeding among patients with portal vein tumoral thrombosis.

We developed a pragmatic bleeding risk-score based on four readily available variables, enabling stratification according to GI bleeding risk during systemic therapy. Although differences in predicted risk across categories were modest, a consistent gradient was observed, reflecting the multifactorial nature of bleeding in advanced HCC. Importantly, the simplicity of the model supports its applicability in routine practice. Although the proposed score is not intended to determine eligibility for a specific systemic therapy, it may assist clinicians in identifying patients requiring more comprehensive bleeding risk assessment before treatment initiation. Patients classified as high risk may benefit from endoscopic reassessment, optimization of primary or secondary prophylaxis according to current portal hypertension guidelines, and closer monitoring during treatment. Future studies should determine whether the score can also inform treatment selection among contemporary systemic therapies. These findings should be interpreted in light of the retrospective, single-center design and require external validation.

Prophylaxis with beta-blockers or banding is highly recommended according to international guidelines from the European Association for the Study of the Liver and the American Association for the Study of Liver Diseases^24^^,^^25^ although most of the literature on risk reduction is based on cirrhotic patients who do not have cancer^13^^,^^26^ This recommendation is also validated in our findings. In cirrhosis patients with high-risk varices, a combination of carvedilol and endoscopic banding has been shown to be more effective than either therapy alone for primary prevention of variceal bleeding^27^ This strategy can be considered in subgroups with higher risk scores in clinical practice, but further validation is needed.

The present study has limitations. First, its retrospective nature may have introduced selection and information biases, particularly regarding the timing and completeness of endoscopic evaluations and data on prophylaxis compliance. Endoscopic characterization of esophagogastric varices (including size and red wale signs) was not uniformly available and some examinations occurred several months earlier; therefore, progression of portal hypertension and esophagogastric varices over time may have resulted in misclassification of baseline bleeding risk in a subset of patients. Another limitation is that baseline endoscopic evaluations performed up to 12-months before treatment initiation were considered representative. Given the dynamic nature of portal hypertension, endoscopic findings may have changed during this interval in some patients, potentially leading to misclassification of bleeding risk. Second, although competing-risk methods were used to account for death, residual confounding cannot be ruled out. Finally, although most bleeding events were confirmed endoscopically, a minority of cases were diagnosed based on predefined clinical criteria when endoscopy was unavailable or inconclusive.

An important consideration when interpreting our findings is that this cohort exclusively included patients treated with first-line sorafenib, reflecting the treatment landscape of the Brazilian public health system during the study period. Since then, the standard of care for advanced HCC has evolved substantially, with immune checkpoint inhibitor-based combinations, particularly atezolizumab plus bevacizumab, becoming preferred first-line therapy. Although the principal predictors identified in our study ‒ esophagogastric varices, splenomegaly, vascular invasion, and previous bleeding ‒ are biologically linked to portal hypertension and therefore are likely to remain clinically relevant irrespective of systemic therapy, the absolute incidence of bleeding and the predictive performance of the proposed score may differ in patients receiving other regimens. Consequently, the score should be regarded as a hypothesis-generating risk stratification tool that requires external validation in contemporary cohorts before routine application across different treatment strategies.

Although similar results have been reported, this topic remains an area of active investigation given the evolving therapeutic landscape of HCC, shifting paradigms in portal hypertension, and the complexity of chronic advanced liver disease, with ongoing challenges in the prevention and management of its complications. The novelty of this work lies in the identification and validation of prognostic factors that should alert physicians managing advanced HCC to the need to stratify bleeding risk and implement measures to mitigate it.

In conclusion, the present study found that upper GI bleeding was a frequent and lethal complication in patients with advanced HCC receiving sorafenib, especially in patients with advanced HCC and altered liver function. Signs of portal hypertension, such as splenomegaly and GI varices, tumor vascular invasion and previous bleeding episodes were key risk factors. These findings highlight the importance of bleeding risk assessment in patients receiving sorafenib and provide a pragmatic framework for future validation in cohorts treated with contemporary systemic therapies.

## Authors’ contributions

Leonardo G. Da Fonseca: Conceptualization; data curation; formal analysis; writing-original draft; writing-review and editing. Lucas Takeshi Ikeoka: Data curation; formal analysis; writing-original draft; writing-review and editing. Eduardo Kim Sampaio: Data curation; formal analysis. Thamires Haick Martins da Silveira, Victor Junji Yamamoto, and Marina Acevedo Zarzar de Melo: Methodology; project administration. Paulo Marcelo Hoff and Jorge Sabbaga: Project administration; resources; supervision; validation; visualization; writing-review & editing. All authors read and approved the final manuscript.

## Ethics approval and consent to participate

The procedures followed were in accordance with the ethical standards of the responsible committee on human experimentation (institutional and national) and with the Helsinki Declaration of 1964 and later versions.

## Patient consent for publication

Not applicable. Consent was waived by the Research Ethics Committee of the University of Sao Paulo School of Medicine (Brazil).

## Use of artificial intelligence tools (to be included only when AI tools are used)

Not applicable.

## Funding

This research did not receive any specific grant from funding agencies in the public, commercial, or not-for-profit sectors.

## Conflicts of interest

Leonardo Da Fonseca: Honoraria: AstraZeneca, Roche, SERVIER, Bayer, MSD. Paulo Marcelo Hoff. Leadership: Oncologia D’Or. Stock and Other Ownership Interests: Oncostar. Honoraria: Bayer Health (I), United medical (I). Consulting or Advisory Role: United Health Group (I), Bayer (I), Lilly (I), Exelixis (I), EMS, AstraZeneca (Inst), EMS. Research Funding: Bayer (Inst), MSD Oncology (Inst), Novartis (Inst), Exelixis (Inst), Roche/Genentech (Inst), AstraZeneca/MedImmune (Inst), Lilly (I), BMS Brazil (Inst), Daiichi Sankyo/Astra Zeneca (Inst).

## Data Availability

The datasets used and/or analysed during the current study are available from the corresponding author on reasonable request.
